# Effectiveness, tolerability and practical application of the newer generation anti-obesity medications

**DOI:** 10.7573/dic.212291

**Published:** 2016-03-08

**Authors:** Jeffrey S. MacDaniels, Thomas L. Schwartz

**Affiliations:** SUNY Upstate Medical University, College of Medicine, Syracuse, NY, USA

**Keywords:** anti-obesity, weight loss, Number Needed to Treat (NNT), Number Needed to Harm (NNH), liraglutide, lorcaserin, orlistat, phentermine, topiramate, bupropion, naltrexone

## Abstract

**Objective:**

Comparison of the efficacy and tolerability of five newer anti-obesity medications to guide clinical decision making, examining bupropion–naltrexone combination, liraglutide, lorcaserin, orlistat, and phentermine–topiramate combination.

**Methods:**

A brief literature review and internet search for high-powered, randomized and placebo-controlled drug trials was conducted. Drug trial information was established for five currently approved anti-obesity medications. Secondarily, a statistical comparison of medications through Number Needed to Treat (NNT) and Number Needed to Harm (NNH) analyses were attempted as a way to provide a clinical analysis across these varied medications. Finally, a commentary about clinical application is issued for each agent accounting for typical side-effects, serious side-effects, mechanism of action and ease of use.

**Results:**

All five agents are currently approved oral medications to lower weight. The NNT range was 3–12, and NNH range was 4–17. The agent with the best NNT is phentermine–topiramate combination (NNT=3) and the agent with the best NNH is bupropion–naltrexone combination (NNH=17).

**Conclusion:**

When considering each patient’s clinical presentation, knowledge of each drug’s mechanism of action, side-effect profile, efficacy, and NNT and NNH values can help in selecting an anti-obesity medication to augment his or her weight loss efforts.

## Introduction

The number of people who are considered overweight or obese is climbing in the United States, with studies now demonstrating that in some demographic and age categories obese persons are beginning to outnumber those who are simply overweight [1]. Many factors contribute to obesity, such as dietary intake, inadequate exercise or sedentary lifestyles, various medical/metabolic conditions, or iatrogenically by medications prescribed to patients. Medications in psychiatry are no exception to this with clear weight-inducing agents such as antipsychotics, antidepressants, and mood-stabilizing antiepileptics and lithium often used in routine care.

Gaining weight is undesirable to healthcare providers as it raises the probability of the development of associated health concerns and medical complications such as diabetes, hypertension, and osteoarthritis. What is sometimes overlooked is the toll it takes on patients’ emotional and psychological state. A few pounds of weight gain may not seem like much, but to the individual patient that experiences unwanted weight gain it may be a larger burden, especially in those with an eating disorder or altered body image. These problems may lead to non-adherence to the prescribed treatment plan, resulting in sub-optimal results or perhaps treatment failure. Unwanted weight gain is a common reason for antidepressant discontinuation and may prompt depressive recurrences in certain individuals.

With a number of treatments and medications available for any particular clinical situation, determining which one to utilize can sometimes be a challenge for the clinician. Clinicians generally become aware of federal approvals and weight loss indications and then assess each drug’s day-to-day side-effect profile and must also determine if there are serious adverse side-effects to contend with. In this manner, each drug is ideally scrutinized prior the clinician prescribing it to any individual patient. Rarely, there are head-to-head clinical trials to guide absolute medication selection. Meta-analyses may be one way to review all weight loss medications as well. Another possible method that can be used to help judge the overall efficacy of medications is through the use a statistical formula known as Number Needed to Treat (NNT) and the same formula when examining undesirable outcomes such as adverse medication effects is the Number Needed to Harm (NNH). Ideally, the NNT is very low, suggesting a better rate of lower weight in the case of weight loss medications. The NNH ideally should be a higher number, suggesting that the medication tends not to create side effects in a majority of patients. A truly ideal drug would have a NNT<NNH ratio that is very high. Clinical data can be obtained for the intervention that is to be examined and can then be considered alongside other interventions and more actively compared. These numbers can be found by taking the inverse of the absolute risk reduction (ARR), which is calculated by subtracting the control event rate (CER) from the experimental event rate (EER), for the placebo and intervention groups, respectively [2]. The NNT or NNH is then rounded up to the nearest whole number and can then be considered in the context of the clinical question. To understand this, consider a theoretical example of an NNT of 14. A NNT of 14 would mean that of one patient for every 14 people undergoing a particular intervention would experience a desired good clinical response attributable to the intervention itself, while the remaining 13 could be due to placebo effects or perhaps to nonresponse itself [2]. The NNH is the number of patients taking a drug that it takes to reach one patient who has an adverse effect or harm. Once the NNT and NNH values are determined, they can then be compared in a tabular or graphical form to allow some comparison among all agents. Lower NNT numbers are often more desirable than higher NNT numbers, with the opposite being true when considering NNH values.

However, there is more to examining these interventions than simply considering low NNT and high NNH numbers as the markers of success. For example, a drug may have an ideal low NNT but may have a rare risk of a serious side-effect (hepatitis, cardiotoxicity). Even a very rare serious side-effect may make prescribing the low-NNT ideal agent less palatable to patient and prescriber alike. Also, ensuring the groups examined in the studies have relatively similar patient backgrounds, baseline characteristics (weight, gender, BMI) and demographics, as well as considering the individual side-effect profiles of each agent and how they relate to the individual patient will help guide the use of these NNT/NNH statistics [3]. This paper seeks to review individual weight loss medications, their mechanism of action, and side-effect profiles, so that clinicians may increase their comfort level in prescribing. Finally, NNT/NNH will be used to make global comparisons regarding these agents.

## Methods

A literature search was conducted and consisted of a two-part approach. First, a brief literature search was performed via Medline up through July 13, 2015. The search terms “obesity treatment and medication” yielded 714 results that were considered to be target papers. Of these, papers of large sample size, statistical power, and randomized controlled design were selected for NNT/NNH review. Lorcaserin, orlistat, and liraglutide, and the combination drugs phentermine–topiramate, and bupropion–naltrexone were specifically searched with this group of available papers, as these represent the most modern era of drugs that are approved as prescription weight loss regimens. This literature review was not intended to be an exhaustive search, rather a source for obtaining background information on the medications themselves and obtaining enough trial data for the purposes of NNT and NNH calculations.

Pharmaceutical companies seeking US Food and Drug Administration (FDA) approval prior to marketing new medications are required to submit a report for review and approval, referred to as a New Drug Application (NDA). NDA applications are required to outline clinical trial information including data on efficacy, safety, adverse effects, and trial sizes. These NDA documents are available on the internet in an unaltered form. Each medication discussed in the paper was found using a basic web search consisting of the “medication name” and the term “NDA”, and this information was secondarily reviewed in order to develop data to conduct NNT/NNH values.

**Figure 1.**
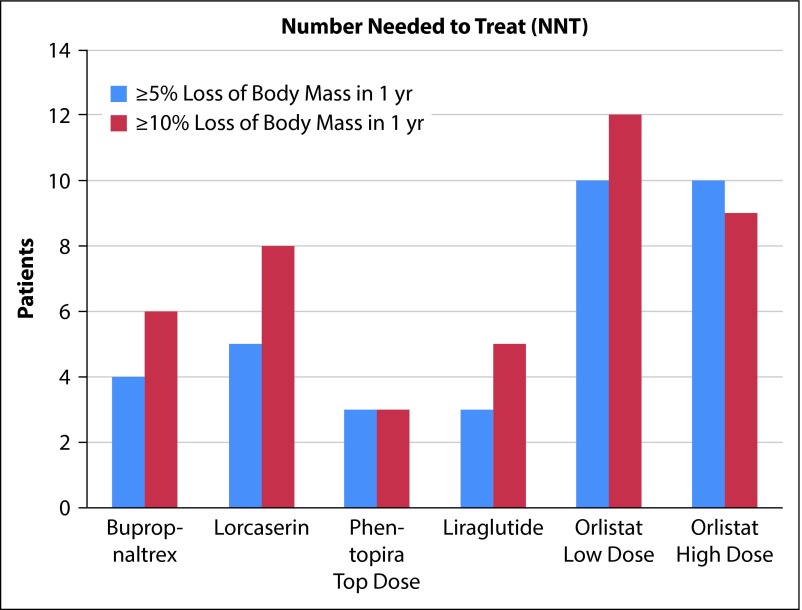
The bar graphs represent the NNT figures for five approved weight loss medications. Lower NNT suggests a greater ability to lower weight. In this figure, phentermine–topiramate combination has the greatest effectiveness results, where orlistat has the least.

**Figure 2.**
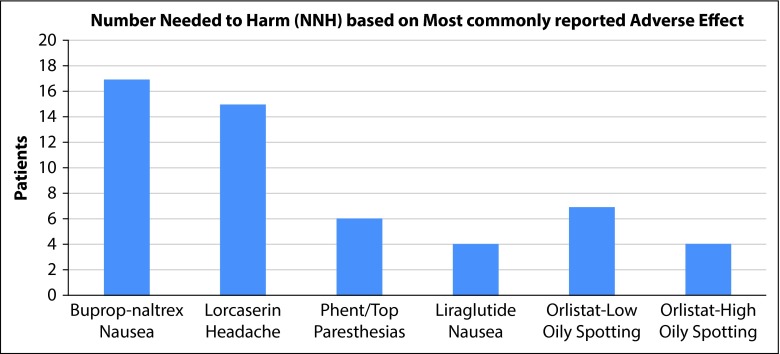
The bar graph represents the NNH figures for five approved weight loss medications. Higher NNT numbers are favorable as this suggests a safer, more tolerable medication. In this figure, the highest NNT and theoretically safest medication in class would be bupropion–naltrexone combination and the least tolerated orlistat and liraglutide.

Many trials defined their goals or end-points as groups that were equal to or greater than 5, 10, and 15% loss of their initial total body mass. As all drugs examined used at least 5 and 10%, those subsets were utilized for this review. The data pertaining to these endpoints were entered into a spreadsheet program, and calculations (described above) were performed to obtain the desired NNT and NNH for each medication [2]. After the NNT and NNH values were calculated by the authors of this article, they were then placed into a graphical format to be used in an efficient manner by a clinician, labeled Figures 1 and 2 in this article.

It should be noted that weight loss studies vary based upon sample size, design (randomized), stratification (results analyzed based upon pre-treatment baseline weight or BMI, etc). Where possible, larger scale, larger sample, randomized and placebo-controlled trials were preferentially utilized in this review. Outside of NNT and NNH outcomes, clinicians must also weigh the general tolerability of the drug and ultimate risk of serious adverse effects even if the chance of these is quite small. For example, the best NNT medication, phentermine–topiramate combination carries a risk of oligohydrosis, acute glaucoma, and acidosis. These are rare events more often detected when topiramate is used for treating epilepsy, but some patients may wish to tolerate a drug with higher NNT to findings to avoid having any of these specific medical risks. Therefore, NNH and NNT is one variable that should be used in clinical decision making analysis and on a patient-by-patient basis.

## Results

### Bupropion–naltrexone combination

The combination bupropion–naltrexone medication is approved for the treatment of obesity. Naltrexone is a mu-opioid receptor antagonist used for management of alcohol dependence and opioid addiction. Bupropion is a norepinephrine-dopamine reuptake inhibitor and is currently used for treatment of both depression and seasonal affective disorder, as well as to aide smoking cessation efforts [4]. Although the mechanism for each individual medication is known, the effect while in their combined role is not entirely clear but could involve naltrexone dampening the neurological reward pathways in the brain, while the bupropion curbs the appetite [4]. Simply, appetite is reduced and the pleasure or reward for eating is also reduced. Due to its bupropion component, this particular weight loss medication is contraindicated in those with a history of seizure disorder or anorexia. The former would be considered a serious adverse event and could limit its use in certain patients. The naltrexone component precludes the use of this medication in those who concurrently take an opiate medication as it could produce a fulminant opioid withdrawal to occur. These withdrawals are not lethal but clearly could be detrimental to the patient or could interfere with a needed pain management regimen.

Data were presented to the FDA in 2010, comparing the naltrexone–bupropion combination (n=2514) medication with placebo (n=1319) in 4 trials [5]. The most common reason for discontinuation in the treatment group was reported as undesirable medication side-effects, while failure to lose weight was attributed to be the leading reason for placebo group dropout [5]. The leading adverse effects attributed to the medication were nausea, headaches and constipation. Two subjects were mentioned in the NDA report as having had a seizure that had no prior history, comprising 0.06% (2/3239) of the total participants. There were three 56-week trials available (NB-301, NB-302, NB-304) with ≥5 and ≥10% end point comparisons between naltrexone SR 32 mg/bupropion SR 360 mg combination medication and placebo groups, which allowed a mean to be calculated for the purposes of NNT and NNH [5]. The experimental group participants that lost ≥5% body mass did so in approximately 53.0%, with the placebo group responding at a rate of 25.9%, yielding an NNT of 4. The experimental group participants that lost ≥10% body mass did so in approximately 28.2%, with the placebo group responding at a rate of 11.1%, resulting in an NNT of 6. The most common adverse effect was nausea, with 6.3% (203/3239) of the pooled medication group reporting this, compared with 0.2% of placebo groups, resulting in an NNH of 17 [5]. Refer to Figures 1 and 2 for NNT and NNH comparisons.

### Lorcaserin

Lorcaserin is a selective serotonin 5-HT2C receptor agonist medication, which may cause an anorectic effect via activation of proopiomelanocortin (POMC) neurons in the hypothalamus [6]. The heart valvulopathies associated with the withdrawal of the nonspecific serotonin receptor medications dexfenfluramine or fenfluramine, are not associated with lorcaserin as this drug’s mechanism is wholly different in nature [6]. As lorcaserin agonizes the 5-HT2C receptor, the possibility of serotonin syndrome serious adverse effects should at least be considered. Typical side effects for lorcaserin include nausea, headaches, fatigue and dizziness [6]. During the BLOSSOM trial, one patient of the 3195 in the 10 mg BID arm during the Behavioral Modification and Lorcaserin Second Study for Obesity Management (BLOSSOM) trial was felt to have symptoms that could be serotonin syndrome [6]. She became vertiginous, developed nausea, vomiting, diarrhea with small amount of blood noted, and blood pressure variability. The serotonin syndrome was likely due to a drug– drug interaction as she was taking the cough suppressant with dextromethorphan. Symptoms did not reappear when the subject restarted lorcaserin one week after antitussive discontinuation [6].

At the end of one year in the BLOSSOM trial, 47.2% (77/1560, *p*<0.001) of the lorcaserin 10 mg twice daily group experienced at least a 5% loss of total body mass, compared with 25% (385/1539, *p*<0.001) who experienced the same level of weight loss in the placebo group [7]. In comparing those that lost at least 10% of their body mass, the lorcaserin 10 mg group experienced a rate of 22.6% (353/1560, *p*<0.001), compared to a response in the placebo group of 9.7% (150/1539, *p*<0.001). This results in a calculated NNT for the ≥5 and ≥10% groups of 5 and 8, respectively. The most common adverse effect experienced were headaches; the lorcaserin group having a 16.8% (537/3195) incidence, the placebo group experiencing 10.1% (321/3185) rate [7], resulting in an NNH of 15. Refer to Figures 1 and 2 for NNT and NNH comparisons.

### Orlistat

Approved by the FDA in 1999 in prescription strength, it was rereleased as an over-the-counter medication a few years later at a lower dose. Orlistat is a reversible direct enzyme inhibitor, particularly of pancreatic and gastric lipases. This prevents triglyceride hydrolysis which results in decreased breakdown and absorption of dietary fat. However this inhibition comes with the risk of adverse side effects such as increased flatulence, oily spotting, greasy loose stools, fecal urgency or frank incontinence [8]. There are no known serious adverse effects, though there may be a worsening of hepatic function early in treatment. This finding is controversial with the most recent data showing little association.

In a study published in 2006, orlistat 60 and 120 mg dosed three times daily was compared against placebo over a period of 6 months [9]. Trial participants with ≥5% loss of body mass included 42.3% (191/452) of orlistat 60 mg, around 44.6% (201/451) of orlistat 120 mg participants, and 23.0% (103/448) of the placebo group. Trial participants with ≥10% loss of body mass included 14.6% (66/452) of orlistat 60 mg, approximately 17.1% (77/451) of orlistat 120 mg participants, and 5.6% (25/448) of the placebo group. This resulted in NNT values of 10 in both medication categories to achieve ≥5% total body mass loss, and an NNT for ≥10% losses in orlistat doses of 60 and 120 mg were 12 and 9, respectively. While these numbers may seem encouraging, it is important to note that a significant portion of participants lost between 0–5% of body mass, with placebo showing 43.3% compared to orlistat 60 and 120 mg of 39.6 and 41.0%, respectively [9]. One of the more common adverse effects reported was oily fecal spotting, which was defined by the sponsor as an “uncontrolled seepage of oil without stool” [9, p. 58]. The orlistat 60 mg group experienced this adverse effect at 17.7% (110/623), the 120 mg group had an incidence of 21.7% (137/632), and placebo group had a 1.1% (7/634) incidence reported [9]. This resulted in an NNH of 7 and 4 for the 60 and 120 mg groups, respectively. Refer to Figures 1 and 2 for further information on orlistat NNT and NNH.

### Phentermine–Topiramate

The combination medication phentermine–topiramate is also approved for obesity treatment. Topiramate is a monosaccharide medication with sulfate substitution. It has been in use since 1996 as an antiepileptic agent, and since 2004 for the prophylactic treatment of migraine headaches. There is some evidence that indicates topiramate can decrease calorie intake through promoting early satiety, or decreases in gluconeogenesis [10]. There are reported neurological side effects of cognitive slowing and paresthesias. More seriously, oligohydrosis, glaucoma, and acidosis may occur. Phentermine was introduced in 1959 for the purposes of weight loss and continues to be prescribed today. Appetite suppression is theorized to be achieved through increased hypothalamic norepinephrine release and raising serum levels of leptin [10]. Phentermine adverse effects include tachycardia, palpitations, insomnia, anxiety, and elevated blood pressure. Although no major congenital malformations were apparent from pregnancies occurring during the clinical trials, there are concerns for this potential. Phentermine and topiramate are labeled with FDA Pregnancy Categories X and D, respectively [10]. The potential for increased teratogenicity would appear to outweigh the benefit of weight loss in a woman trying to become pregnant, so the combination medication was assigned Pregnancy Category X [11].

Two trials in particular were 56 weeks in duration. OB-302 examining low and high doses, while OB-303 investigated middle and high doses [10]. This allows for comparison of the top dose of phentermine/topiramate, 15/92 mg daily between the trials but, limits the ability of examining the second drug dosage in each trial. The mean values of the top dose in OB-302 and OB-303 for ≥5 and ≥10% loss of total body mass were approximately 68.4 and 47.4%. This is compared to the placebo group losses for ≥5 and ≥10% loss of total body mass of 19.1 and 7.4%, which results in NNT values of 3 for both weight loss end points. The most common adverse effect of paresthesias [11] occurred in 19.9% (315/1580) of the high dose medication group, the placebo group experienced a much lower rate of 1.9% (30/1561), resulting in an NNH for this particular medication of 6 [10]. Refer to Figures 1 and 2 for NNT and NNH comparisons.

### Liraglutide

The most recent anti-obesity medication approved is the glucagon-like peptide-1 (GLP-1) receptor agonist liraglutide, an endogenous ligand [12]. GLP-1 receptors are located on pancreatic beta cells, the activation of which causes more insulin to be released and the suppression of glucagon to occur secondarily. This results in decreased appetite and promotes less energy storage [12]. The medication as a once daily 1.8 mg subcutaneous injection has been used in the United States since 2010 as a treatment for type 2 diabetes mellitus, and the 3 mg once daily subcutaneous version was FDA approved in 2014 for the treatment of obesity. It was approved in 2015 by the European Medical Agency (EMA) for the same purpose [13]. There is a theoretical increased risk of medullary thyroid carcinoma based on studies in rodents, but no known case have been reported in humans. This is the only non-oral drug discussed and this drug requires patients to inject their medication.

Several trials were completed with the largest being Trial 1839, a 56-week Phase 3 trial which compared weight loss with liraglutide 3 mg (n=2437) against placebo (n=1225) [12]. Approximately 63% (1536/2432) of the experimental group achieved a weight loss of ≥5% body mass, and 27.1% (331/1220) of the placebo group achieved the same loss of body mass, resulting in an NNT of 3. Approximately 33.1% (805/2432) of the patients in the experimental group achieved a loss of ≥10% of body mass, while 10% (129/1290) of the placebo group achieved this same end point resulting in an NNT of 5. Around 40% of trial participants reported adverse effects of nausea, 15% had vomiting, and 21% experienced diarrhea, compared to patients in the placebo group at 14, 4, and 10%, respectively [12]. In considering the most common adverse effect of nausea, the liraglutide group experienced a rate of 39.3% (1329/3384), while the placebo group experienced the nausea at a rate of 13.8% (267/1941), resulting in an NNH of 4. In these trials, 9 patients developed a serious or severe case of acute pancreatitis compared to one in the placebo group [12]. An RCT study was written by Pi-Sunyer et al. in 2015, the data of which confirm the results of earlier trials [14]. Refer to Figures 1 and 2 for NNT and NNH comparisons.

## Discussion

Trying to determine which anti-obesity medication may be appropriate for a particular patient can be dependent on several factors. Cost is a major consideration for many patients. Some insurance plans are beginning to cover these medications, but other plans do not consider them to be necessary or to be “cosmetic” in nature, including the U.S. Medicare system which has specific exclusionary language regarding this topic. Incurring out-of-pocket expenses might make this line of medications harder to afford, as many are still on patent with no generic available. Some insurers insist the patient enroll in commercially available diet and exercise programs, which also carries a financial burden. However, for each of these medications, an educational and behavioral approach is generally suggested and warranted for optimum weight loss results.

The effectiveness of medications is another important factor to consider. Does this medication work, and if so, how well? To answer this, a comparison of the Number Needed to Treat (NNT) for the medications in question is warranted. In examining their efficacy over a 56-week period, the NNT ranged from 3 patients for some of the newest medications such as liraglutide, bupropion–naltrexone, and phentermine–toperimate, and as high as 10–12 for the older orlistat. While this is not a staggering difference, it gives a framework as to the relative effectiveness of each agent. It may take a prescriber 4 patients on bupropion–naltrexone combination to see significant weight loss comparable to its regulatory studies, where it may take 12 orlistat patients to see the same results. In foreshadowing to the next paragraph about tolerability, the more effective combination preparation could be detrimental or lethal to a patient with known epilepsy.

Tolerability is the next point of discussion for these medications. Adverse effects largely included gastrointestinal side-effects such as nausea, diarrhea, constipation, abdominal pain, oily spotting or frequent defecation. Rarer neurological effects are noted with phenteramine–topiramate and bupropion–naltrexone, such as paresthesias and headaches. Liraglutide had pancreatitis as a rare adverse effect. Felt to be safer and better tolerated because of their higher NNH figures were bupropion–naltrexone (nausea, NNH=17), and lorcaserin (hyperglycemia, NNH= 13; nausea, NNH=15). Orlistat and liraglutide were noted for low NNH figures, with oily spotting (NNH=4) and nausea (NNH=4) causing more intolerability and dropout, respectively. Generally, gastrointestinal distress is not a serious or lethal side-effect but is highly intolerable to patients. Orlistat may have some of the least risky serious adverse effects, but its notoriety for gastrointestinal distress extremely lowers its NNH, making it an unfavorable medication.

Based on these figures, some treatments appear more favorable than the others. Bupropion–naltrexone had a fairly low NNT to achieve either 5 or 10% loss of body mass, with a NNH based on adverse effects higher than the rest of the medications considered in this review. It may have the best effectiveness/tolerability ratio. For the same reasons, phentermine–topiramate is similarly rated. However, these two medications may have rarer but significant serious adverse risks to consider on a patient-by-patient basis. Liraglutide appears somewhat favorable based on NNT, but the lower NNH that resulted from reported nausea may decrease patients’ desire to continue taking the medication. Lorcaserin may not be as efficacious as some of the previously listed medications, but it does have the second highest NNH figure making it one of the safer and more tolerable option for many patients. Orlistat appears to have the most difficulties, with low NNHs and high NNTs.

## Conclusion

With the population becoming more overweight over time, the topic of pharmacologic augmenting of failed diet and exercise treatments is both timely and necessary. The increased prevalence of type 2 diabetes mellitus, cardiovascular disease, fatigue, depression, premature osteoarthritis, feelings of poor self-image or worth, and other associated risks of obesity are all reasons to promote pharmacologic management of obesity. While it may be stoically sanctioned to discuss “tough love” and insist that all weight loss can be achieved through “willpower, a sensible diet and exercise,” this is sometimes not enough. For patients that cannot exercise (enough or at all), or have issues controlling their appetite, medications that can aid in their weight loss reduction is clinically warranted.

This paper would suggest that all approved medications have the ability to lower weight gain from metabolic, medical, or even iatrogenic causes. All agents carry common and more esoteric or extreme side-effects and these must be considered when prescribing. Based solely on NNT/NNH harm analyses, bupropion–naltrexone and phentermine–topiramate combinations may have a clinical advantage.

## Abbreviations:

5-HT2C: serotonin 2C receptor;
ARR: absolute risk reduction
BLOSSOM: behavioral modification and lorcaserin second study for obesity management
CER: control event rate
EER: experimental event rate
EMA: European medical agency
FDA: food and drug administration
GLP-1: glucagon-like peptide-1
NDA: new drug application
NNH: number needed to harm;
NNT: number needed to treat
RCT: randomized control trial.
